# Is the Lactate/Albumin Ratio Associated with 28-Day Mortality in Critically Ill Patients That Underwent Open Gastric Cancer Surgery? A Retrospective Single-Center Study

**DOI:** 10.3390/jcm15093345

**Published:** 2026-04-28

**Authors:** Yavuz Selim Kahraman, Veysel Garani Soylu, Öztürk Taşkın

**Affiliations:** 1 Department of General Surgery, Division of Surgical Oncology, Kastamonu Training and Research Hospital, Kastamonu 37150, Türkiye; 2 Department of Intensive Care, Kastamonu University, Kastamonu 37150, Türkiye; vgsoylu@hotmail.com; 3 Department of Anesthesiology and Reanimation, Kastamonu University, Kastamonu 37150, Türkiye; drozturk275@hotmail.com

**Keywords:** gastric cancer, 28-day mortality, lactate/albumin ratio, intensive care

## Abstract

**Objectives:** The aim of this study is to investigate the relationship between the lactate/albumin ratio (LAR) and 28-day mortality in gastric cancer patients undergoing monitoring in a postoperative intensive care unit due to reasons such as haemodynamic instability, need for vasopressor support, or intraoperative bleeding. **Methods:** This retrospective study included patients followed up at the tertiary surgical intensive care unit of Kastamonu University Faculty of Medicine between January 2020 and October 2025 who were diagnosed with histologically confirmed gastric adenocarcinoma and underwent total open surgery or subtotal gastrectomy + D2 lymphadenectomy. The patients were categorized into two groups: non-survivors within 28 days (*n*: 45) and survivors within 28 days (*n*: 139). **Results:** A total of 184 critically ill patients (110 males, 74 females) who underwent gastric adenocarcinoma surgery and were followed up in the surgical intensive care unit were included in this study. The mean age of the patients was 72.2 ± 11.3 years. Of these patients, 139 (75.5%) were survivors, and 45 (24.5%) were non-survivors. Albumin, the C-reactive protein (CRP)/albumin ratio, lactate, and the lactate/albumin ratio were associated with 28-day mortality. Receiver operating characteristic (ROC) analysis showed that the LAR (area under the curve (AUC): 0.839) was superior to the serum albumin (AUC: 0.736) and lactate levels (AUC: 0.796) for predicting 28-day mortality. The optimal cut-off value of the LAR was 0.82, and an LAR of ≥ 0.82 was shown to be a significant and independent prognostic factor for 28-day mortality in patients with stomach cancer in a critical postoperative condition (odds ratio (OR): 4.78, confidence interval (CI): 1.09–21.08, *p* = 0.0386). **Conclusions:** The lactate/albumin ratio is a prognostic parameter for 28-day mortality in critically ill postoperative gastric cancer patients. The optimal cut-off value for the lactate/albumin ratio is 0.82.

## 1. Introduction

Stomach cancer is one of the most common cancers worldwide and is a leading cause of cancer-related mortality [1]. The tumour–node–metastasis (TNM) classification system is generally used to determine the prognosis of stomach cancer. However, parameters such as age, tumour location, and lymphovascular invasion also influence the prognosis of gastric cancer [2]. In addition to these parameters, inflammation parameters such as lactate dehydrogenase and albumin have been associated with patient prognosis in gastric cancer and many other types of cancers [3,4].

Lactate is a marker of tissue hypoperfusion and metabolic stress and can predict disease severity. The relationship between lactate and mortality in critical illness has been confirmed in previous studies [5]. Due to its prognostic role, lactate has been widely used in critically ill patients for screening, diagnosis, risk stratification, and follow-up [6]. Lactate levels in critically ill patients are strongly correlated with Sequential Organ Failure Assessment (SOFA) scores and outcomes [7]. In the literature, there are many studies on the prognostic importance of lactate in patients with sepsis, trauma, and shock, but information on its prognostic importance in gastric cancer is limited [8].

Albumin is a protein produced in the liver that possesses anti-inflammatory, antioxidant, and antithrombotic properties [9]. Although albumin is a protein indicative of malnutrition, it is a negative acute-phase reactant in critical illness conditions. Low serum albumin levels have been associated with increased mortality in patients with cardiovascular conditions, kidney disorders, COVID-19, and cancers [10]. There is a non-linear relationship between albumin levels and cancer mortality risk.

The lactate/albumin ratio (LAR), which is derived from the ratio of albumin, an important prognostic marker in cancer patients, to lactate, an important prognostic marker in critically ill patients, has been investigated in terms of prognosis in diseases such as COVID-19, pneumonia, and acute respiratory distress syndrome [11]. However, there are no studies in the literature focusing on critically ill postoperative gastric cancer patients and investigating the effect of LAR on 28-day mortality. For these reasons, in this study, we aimed to investigate the relationship between LAR and 28-day mortality in gastric cancer patients who underwent surgery and were followed up in the postoperative intensive care unit (ICU) as critical patients due to reasons such as haemodynamic instability, need for vasopressor support, or intraoperative bleeding.

## 2. Materials and Methods

### 2.1. Study Design and Setting

This retrospective study included patients followed up at the tertiary surgical intensive care unit of Kastamonu University Faculty of Medicine between January 2020 and October 2025 who were diagnosed with histologically confirmed gastric adenocarcinoma and underwent total open surgery or subtotal gastrectomy + D2 lymphadenectomy. Ethical approval for this study was obtained from the Kastamonu University Faculty of Medicine Non-Interventional Clinical Research Ethics Committee (date: 18 December 2025; approval number: 2025-101). This study was conducted in accordance with the principles of the Helsinki Declaration (1964). Due to the retrospective design of this study and the nature of data collection, informed consent was not deemed necessary.

### 2.2. Study Population

**Figure 1:**
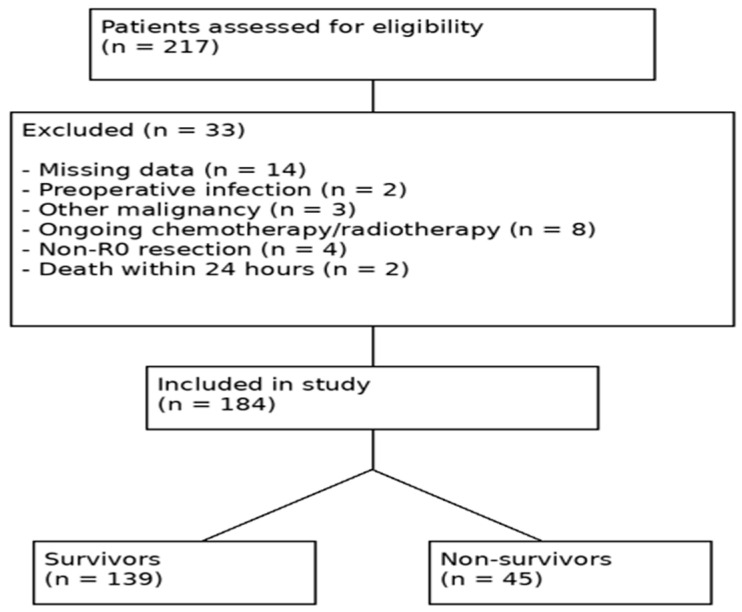
The flow diagram of patient selection, including the number of patients assessed for eligibility, excluded cases with reasons, and the final study population.

According to the inclusion and exclusion criteria, 184 critically ill patients who underwent gastric adenocarcinoma surgery and were followed up in the surgical intensive care unit were included in this study by performing a retrospective search of the hospital information management system to obtain complete data. Patients with missing data (*n* = 14) were excluded during the screening process, and only complete cases were included in the final analysis. The patient selection process is summarized in Figure 1.

### 2.3. Inclusion and Exclusion Criteria

The inclusion criteria include the following: an age of 18 years or older, a histologically confirmed diagnosis of gastric adenocarcinoma, having undergone R0 resection, and being monitored in the surgical intensive care unit due to postoperative haemodynamic instability (requiring vasopressor support). The exclusion criteria include the following: missing data, active infection in the preoperative period, presence of other malignancies, ongoing chemotherapy or radiotherapy treatment, presence of hematological or systemic immunological diseases, death within 24 h postoperatively, and surgical resection other than R0 resection. Patients who died within the first 24 h postoperatively were excluded to avoid the confounding effects of early mortality primarily related to intraoperative or immediate postoperative complications.

### 2.4. Data Collection

Patients’ demographic characteristics, intensive care and hospital stay days, comorbidities, and preoperative 24 h blood sample values for leukocytes, neutrophils, platelets, lymphocytes, monocytes, CRP, and albumin were recorded. Upon admission to the postoperative intensive care unit, APACHE II score, SAPS II, and lactate values based on arterial blood gas were recorded. Lactate levels were obtained from arterial blood gas samples taken within the first hour following admission to postoperative intensive care. Preoperative albumin levels were used to reflect baseline physiological and nutritional status, whereas postoperative lactate levels obtained at ICU admission were used to reflect acute physiological stress.

Due to albumin being a negative acute-phase reactant, its preoperative value [normal albumin range in blood is 3.5 to 5.4 g (g/dL)] and its postoperative lactate value upon admission to the intensive care unit were used in this study. The CRP value was divided by the albumin value to calculate the CRP/albumin ratio (CAR), and the lactate value was divided by the albumin value to calculate the lactate/albumin ratio. Postoperative 28-day mortality data were obtained from medical records or through telephone follow-up. Based on these outcomes, patients were categorized into two groups: non-survivors within 28 days (*n*: 45) and survivors within 28 days (*n*: 139).

### 2.5. Statistical Analysis

The statistical analyses of this study were performed using IBM SPSS Statistics (version 26.0). The distribution suitability of continuous variables was assessed using the Kolmogorov–Smirnov test. Continuous variables that followed a normal distribution were presented as mean ± standard deviation, and a Student’s *t*-test was used for comparisons between two groups. Categorical variables were expressed as numbers and percentages; the chi-square (χ^2^) test or Fisher’s exact test, when appropriate, was applied for group comparisons. The predictive performance of serum lactate level, albumin level, and lactate/albumin ratio for 28-day mortality was assessed using ROC curve analysis, and AUC (area under the curve) values were reported. The optimal cut-off points were determined using the Youden index (sensitivity + specificity − 1). To identify risk factors for mortality, univariate logistic regression analysis was first performed, and then significant parameters were included in a multivariate logistic regression model. The significant number of variables included in the multivariable model was limited to avoid overfitting.

Results were presented as odds ratios (ORs), 95% confidence intervals, and *p*-values. Furthermore, Pearson correlation analysis was performed with APACHE II score and SAPS II to evaluate the relationship between lactate/albumin ratio and clinical severity; correlation coefficients (r) and *p*-values were reported. All tests with *p* < 0.05 were considered statistically significant. Based on the literature, the prognostic value of the lactate/albumin ratio in critically ill patients was determined using a moderate effect size (Cohen’s d = 0.5), 80% power, and a two-sided significance level of 0.05%, resulting in a minimum required sample size of 128 patients. Considering a potential 20% missing data point, the target sample size was set at a minimum of 160. For this study, 184 patients constitute a sufficient sample size.

## 3. Results

### 3.1. Baseline Characteristics of the Overall Study Population

The number of patients included in this study was 184. A total of 110 (59.8%) patients were male, and 74 (40.2%) patients were female. The mean age of the patients was 72.20 ± 11.33 years. Overall, 138 (75.0%) patients were aged 65 years and older, and 46 (25.0%) patients were under 65 years of age. A total of 143 (77.7%) patients had at least one comorbidity. The average APACHE II score of patients admitted to the intensive care unit was 21.24 ± 7.05, and the average SAPS II was 41.24 ± 16.56. The average intensive care unit stay for all patients was 6.49 ± 9.96 days, and the average hospital stay was 13.35 ± 14.81 days. Overall, 45 (24.5%) patients were in the group that died within 28 days after surgery (Table 1).

### 3.2. Comparison of the Baseline Clinical Characteristics Between Survivors and Non-Survivors

According to the comparative statistical analysis of laboratory parameters between groups, the following parameters were significant for 28-day mortality: hemoglobin (10.30 vs. 11.20 g/dL, *p* = 0.0383), hematocrit (32.00 vs. 34.60%, *p* = 0.0454), thrombocytes (197 ± 92.18 vs. 239 ± 111.11 10^3^/μL, *p* = 0.0021), creatinine (0.97 ± 1.25 vs. 0.80 ± 1.00 mg/dL, *p* = 0.0267), albumin (2.40 ± 0.67 vs. 2.90 ± 0.62 g/dL, *p* = <0.0001), the CRP/albumin ratio (48.33 ± 47.98 vs. 18.85 ± 44.91, *p* = 0.0429), lactate (2.50 ± 1.47 vs. 1.40 ± 0.85 mmol/L, *p* < 0.0001), and the lactate/albumin ratio (1.05 ± 0.79 vs. 0.48 ± 0.35, *p* < 0.0001). In the non-survivor group, creatinine, CRP/albumin ratio, lactate, and lactate/albumin ratio values were higher, while hemoglobin, hematocrit, platelet, and albumin values were lower (Table 2).

### 3.3. Mortality Prediction Performance of Lactate, Albumin, and Lactate/Albumin Ratio

**Figure 2:**
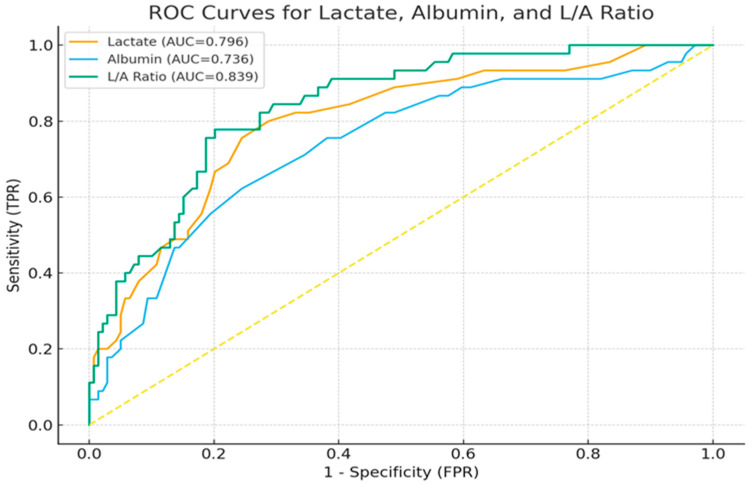
The ROC analysis of lactate/albumin ratio, serum lactate level, and serum albumin level for predicting 28-day mortality.

We performed ROC analysis to predict 28-day mortality and to find the optimal cut-off value of the LAR for determining the 28-day mortality. ROC analysis showed that the LAR (AUC: 0.839, *p* < 0.001) was superior to the serum albumin (AUC: 0.736, *p* < 0.001) and lactate levels (AUC: 0.796, *p* < 0.001) in predicting 28-day mortality. In addition, the optimal cut-off value of the LAR was found to be 0.82 (Figure 2) (Table 3).

### 3.4. Comparison of the Baseline Clinical Characteristics Between Patients with LAR ≥ 0.82 and Patients with LAR < 0.82

After determining the LAR cut-off value, the overall study population was divided into two groups: patients with LAR ≥ 0.82 and patients with LAR < 0.82. There were statistically significant differences between the groups in terms of the mortality (55.6% vs. 8.3%, *p* < 0.001), SAPS II (47.78 ± 18.90 vs. 42.46 ± 15.78, *p* = 0.003), APACHE II score (26.29 ± 8.08 vs. 21.72 ± 7.01, *p* = 0.004), platelet count (197 ± 104.6 vs. 242 ± 105.7 10^3^/μL, *p* = 0.002), lymphocyte count (0.84 ± 0.78 vs. 1.27 ± 0.93 10^3^/μL, *p* = 0.001), BUN value (33 ± 42.2 vs. 41 ± 48.1, *p* = 0.047), albumin (2.40 ± 0.65 vs. 2.90 ± 0.60 g/dL, *p* < 0.001), CRP/albumin ratio (46.80 ± 50.90 vs. 18.12 ± 42.50, *p* = 0.027), and lactate (2.90 ± 1.16 vs. 1.30 ± 0.46 mmol/L, *p* <0.001) values (Table 4).

**Figure 3:**
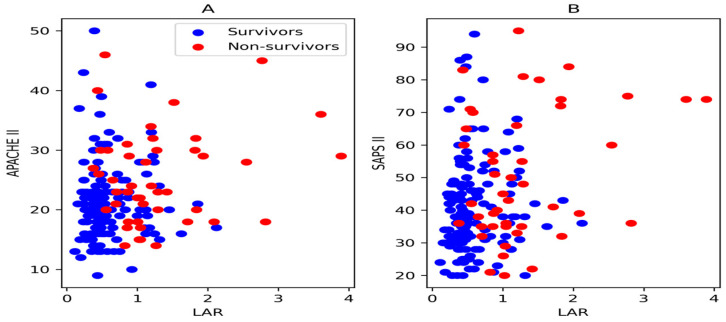
The Pearson correlation analysis of LAR with the (**A**) APACHE (Acute Physiology and Chronic Health Evaluation) II score and (**B**) SAPS (Simplified Acute Physiology Score) II.

According to Pearson correlation analysis, a positive weak correlation was found between the SAPS II and the LAR (r = 0.260, *p* < 0.001). There was a limited correlation between APACHE II score and the LAR (r = 0.205, *p* = 0.005) (Figure 3).

### 3.5. Regression Analysis of the Patients

In the univariate and multivariate regression analyses, an LAR of ≥ 0.82 was shown to be a significant and independent prognostic factor for 28-day mortality in patients with stomach cancer in a critical postoperative condition (odds ratio: 4.78, CI: 1.09 –21.08, *p* = 0.0386) (Table 5).

### 3.6. Pairwise Comparison of ROC Curves Using the DeLong Test

In Table 6, the differences between the ROC curves obtained for lactate and albumin are compared using the DeLong test. The DeLong test is a non-parametric method used to evaluate whether the AUC difference between two dependent ROC curves is statistically significant. A *p*-value of <0.05 is considered statistically significant. A comparison of AUC differences and *p*-values is provided in Table 6.

### 3.7. Distribution of Lactate/Albumin Ratio (LAR) in Survivors and Non-Survivors

**Figure 4:**
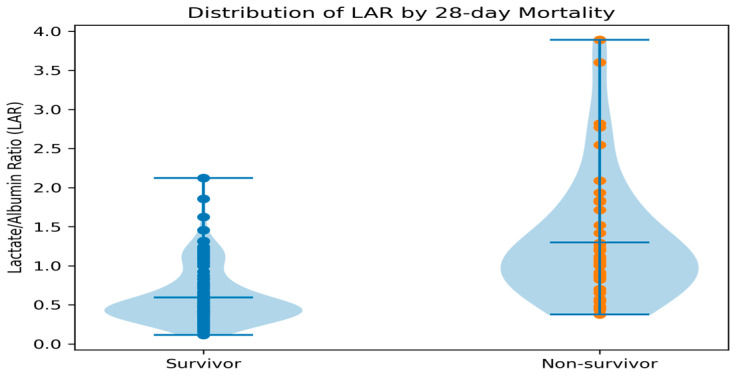
Distribution of lactate/albumin ratio (LAR) in survivors and non-survivors.

Figure 4 shows a violin plot illustrating the distribution of lactate/albumin ratio (LAR) values between survivors and non-survivors. Each data point is overlaid to provide a clearer visualization of the distribution and variability within each group. This additional visualization supports the statistical findings by clearly showing the difference in LAR values between the two groups.

## 4. Discussion

This study has demonstrated that age, APACHE II score, and SAPS II are significant parameters for 28-day mortality in critically ill patients who have undergone surgery for gastric cancer. Additionally, it was found that albumin, lactate, and the lactate/albumin ratio, each individually, are significant for 28-day mortality. Among these three parameters, the lactate/albumin ratio had the highest AUC value (AUC: 0.839, *p* < 0.001). A lactate/albumin ratio of ≥0.82 is a significant and independent prognostic factor for 28-day mortality, which is one of the important findings of this study.

Lactate is a blood parameter that appears after tissue hypoxia and rises due to causes such as impaired glucose metabolism and catecholamine use [12,13]. Lactate is an important prognostic marker in sepsis patients; however, it is also associated with increased metastasis, tumour recurrence, and poor outcomes in cancer patients [14,15]. High lactate is associated with surgical stress. A study by Yang et al. demonstrated the significant effect of the gastrointestinal surgical site on postoperative lactate levels. In this study, the highest lactate values after oesophageal surgery were observed in patients who underwent gastric surgery [16]. According to this study, the mean lactate value in patients who underwent gastric surgery was 2.3 mmol/L. In our study, the mean lactate value was 1.60 mmol/L, but the mean lactate value in the non-survivor group was 2.50 mmol/L, which was higher than that in the survivor group. Additionally, elevated lactate levels were associated with 28-day mortality (cut-off: 1.9 mmol/L, *p*: <0.001, AUC: 0.796).

Albumin is an important indicator of nutritional status and inflammation. Additionally, it is a negative acute-phase reactant in acute inflammatory conditions such as trauma, burns, and acute infections [17]. Serum albumin levels are a strong prognostic indicator of cancer-related mortality in patient cohorts and the general population [18]. In a study involving colorectal cancer patients, a 0.1 g/dL increase in albumin levels was associated with a 7.3% reduction in morbidity and a 15.6% reduction in mortality [19]. In another study of patients with cardia adenocarcinoma, patients with normal albumin levels had a higher 5-year survival rate compared to those with abnormal albumin levels [20]. Serum albumin is an important component of certain cancer scoring systems and acts as a balancing factor in cancer prognosis. The C-reactive protein/albumin ratio has been shown to predict adverse outcomes in colorectal cancer, oral squamous cell carcinoma, gallbladder cancer, lung cancer, and thoracic oesophageal cancer [18,21]. Due to albumin being a negative acute-phase reactant, we used the albumin value measured within 24 h preoperatively in our study, and the mean albumin value was 2.80 g/dL. The mean albumin level was lower in the survivor group (2.40 g/dL). According to the multiple regression analysis, an albumin level below 2.5 g/dL was a parameter associated with 28-day mortality (AUC: 0.736, *p*: <0.001). In the multivariate regression model, the low significance of lactate alone and the high significance of the lactate/albumin ratio support the use of the lactate/albumin ratio as a more robust prognostic marker in critically ill postoperative patients. Preoperative albumin and postoperative lactate may reflect different physiological domains; however, their combination in the LAR may provide a more comprehensive assessment of both baseline reserve and acute stress response.

The lactate/albumin ratio is a combination of two important parameters in terms of prognosis in critically ill patients. Especially in cancer patients, lactate and albumin are valuable prognostic parameters when considered separately. Studies indicate that the lactate/albumin ratio may serve as a prognostic marker in conditions such as sepsis, coronary artery disease, and acute respiratory distress syndrome [22,23,24,25]. However, the lactate/albumin ratio has not been investigated in terms of the prognosis of critically ill patients who have undergone surgery for cancer, especially stomach cancer, and there is limited information available in the literature on this topic.

In a study, a lower lactate/albumin ratio was associated with higher survival rates. Its authors stated that the optimal cut-off value for the lactate/albumin ratio was 0.57 and that it had 63% sensitivity and 73% specificity in predicting intensive care unit mortality [11]. In a study conducted by Acharya and colleagues in 2025, the prognostic importance of the lactate/albumin ratio in respiratory failure and sepsis was investigated; they reported that the threshold value of the lactate/albumin ratio for predicting mortality in sepsis patients was 1.78, and the AUC value was 0.914. In the same study, they stated that, in acute renal failure cases, the lactate/albumin ratio had a threshold value of 1.978 for mortality and an AUC value of 0.878 [23]. As there was no study in the literature similar to ours, a complete comparison with the literature could not be made, but according to our study, a threshold value of 0.82 and an AUC value of 0.839 were found for 28-day mortality. Rather than evaluating lactate and albumin alone for prognosis, evaluating the lactate/albumin ratio yielded a higher AUC value and better sensitivity and specificity values (for the lactate/albumin ratio: sensitivity: 0.778; specificity: 0.799). The two important prognostic parameters in intensive care patients are the APACHE II score and SAPS II [26]. While some baseline clinical parameters such as BMI and ASA classification were unavailable, disease severity was assessed using the APACHE II score and SAPS II. These scores are well defined and widely used parameters in predicting mortality in intensive care patients. In a study conducted by Wnag B and colleagues in patients with sepsis and septic shock, a positive correlation was found between the lactate/albumin ratio and the APACHE II score and SAPS II [27]. Similarly, the LAR was found to be superior in predicting mortality compared to lactate or albumin values alone. The LAR and APACHE II scores were correlated in patients with pneumosepsis [15]. In our study, there was also a weak positive and significant correlation between the lactate/albumin ratio and the APACHE II score and SAPS II (for APACHE II: r = 0.205, *p* = 0.005; for SAPS II: r = 0.260, *p* < 0.001). According to the DeLong test results, the AUC value of LAR is statistically significantly higher than that of both lactate and albumin. In contrast, no statistically significant difference was found between lactate and albumin.

Clinically, the lactate/albumin ratio can help identify high-risk patients early on. This can enable closer hemodynamic monitoring and timely interventions to prevent postoperative complications. In future studies, the inclusion of additional biomarkers could further improve risk stratification and enhance preoperative decision-making. In addition to molecular markers, new technologies have the potential to improve both clinical decision-making processes and training experiences in complex gastrointestinal surgeries [28,29].

### Limitations

This study has several limitations: the study design was retrospective; long-term follow-up data were unavailable; and it was a single-centre study. Due to the retrospective nature of this study, the absence of certain descriptive clinical variables, such as steroid use, ASA Physical Status Classification Grade classification, Body Mass Index (BMI), and postoperative complications, may also constitute a limitation. Due to the retrospective nature of the study, Cox regression analysis could not be performed, but this could be implemented in future studies to strengthen survival analyses.

## 5. Conclusions

The lactate/albumin ratio is a prognostic parameter for 28-day mortality in critically ill postoperative gastric cancer patients. The optimal cut-off value for the lactate/albumin ratio is 0.82. In this patient group, the lactate/albumin ratio is a more valuable prognostic parameter than lactate or albumin alone. It also shows a positive correlation with prognostic scoring systems such as APACHE II and SAPS II.

## Figures and Tables

**Table 1:** Baseline characteristics of patients: survivors and non-survivors.

Population Characteristics	Overall (*n* = 184)	Survivors (*n* = 139, 75.5%)	Non-Survivors (*n* = 45, 24.5%)
Male, *n* (%)	110 (59.8)	89 (64.0)	21 (46.7)
Female *n* (/%)	74 (%40.2)	50 (%36.0)	24 (%53.3)
Age (mean ± SD)	72.20 ± 11.33	70.58 ± 10.77	77.22 ± 11.66
≥65 years (*n*/%)	138 (%75.0)	99 (%71.2)	39 (%86.7)
<65 years (*n*/%)	46 (%25.0)	40 (%28.8)	6 (%13.3)
Comorbidities (*n*/%)	143 (%77.7)	108 (%75)	35 (%25)
Diabetes mellitus (*n*/%)	21(%11.4)	19 (%90)	2 (%10)
Hypertension (*n*/%)	42 (%22.8)	33 (%78)	9 (%22)
Cerebrovascular accident (*n*/%)	17 (%9.2)	14 (%82)	3 (%18)
Chronic renal failure (*n*/%)	10 (%5.4)	8 (%80)	2 (%20)
Cardiac failure (*n*/%)	32 (%17.3)	26 (%81)	6 (%19)
Respiratory failure (*n*/%)	21 (%11.4)	16 (%76)	5 (%24)
APACHE II (mean ± SD)	21.24 ± 7.05	20.55 ± 6.51	25.40 ± 7.82
SAPS II (mean ± SD)	41.24 ± 16.56	39.0 ± 14.85	50.89 ± 19.79
Hospital admission day (mean ± SD)	13.35 ± 14.81	14.19 ± 16.15	10.73 ± 9.24
ICU admission day (mean ± SD)	6.49 ± 9.96	6.27 ± 10.48	7.16 ± 8.22

Abbreviations: APACHE: acute physiology and chronic health evaluation. SAPS: simplified acute physiology score. ICU: intensive care unit.

**Table 2:** The baseline laboratory parameters of the patients: survivors vs. non-survivors.

Patient Parameters	Overall (*n* = 184) (Mean ± SD)	Survivors (*n* = 139) (Mean ± SD)	Non-Survivors (*n* = 45) (Mean ± SD)	*p*-Value
Hemoglobin, g/dL	10.80 (±2.09)	11.20 (±2.06)	10.30 (±2.12)	0.0383 *
Hematocrit %	33.55 (±6.10)	34.60 (±5.92)	32.00 (±6.40)	0.0454 *
Leukocytes, 10^3^/Μl	10.11 (±7.72)	10.11 (±7.66)	10.10 (±7.98)	0.9661
Neutrophils, 10^3^/Μl	7.94 (±6.18)	8.00 (±5.82)	7.87 (±7.20)	0.4210
Platelets, 10^3^/Μl	230 (±108.94)	239 (±111.11)	197 (±92.18)	0.0021 *
Lymphocytes, 10^3^/μL	1.08 (±0.89)	1.17 (±0.85)	0.80 (±1.02)	0.1565
Monocytes, 10^3^/μL	0.60 (±0.60)	0.60 (±0.64)	0.62 (±0.48)	0.7698
BUN, mg/dL	35 (±44.73)	33 (±44.49)	48 (±44.73)	0.1217
Creatinine, mg/dL	0.84 (±1.08)	0.80 (±1.00)	0.97 (±1.25)	0.0267
CRP, mg/dL	70.9 (±110.38)	58.0 (±112.55)	117.0 (±102.63)	0.2042 *
Albumin, g/dL	2.80 (±0.67)	2.90 (±0.62)	2.40 (±0.67)	<0.0001 *
CRP/albumin ratio	29.20 (±46.11)	18.85 (±44.91)	48.33 (±47.98)	0.0429 *
Lactate, mmol/L	1.60 (±1.17)	1.40 (±0.85)	2.50 (±1.47)	<0.0001 *
Lactate/albumin ratio	0.55 (±0.58)	0.48 (±0.35)	1.05 (±0.79)	<0.0001 *

Abbreviations: BUN: blood ürea nitrogen. CRP: C-reactive protein. * Statistically significant (*p* < 0.05).

**Table 3:** Values of AUC, sensitivity, and specificity for serum lactate, serum albumin, and the lactate/albumin ratio for predicting 28-day mortality.

	AUC	95% CI	Cut-Off **	Sensitivity	Specificity	*p*-Value
Lactate, mmol/L	0.796	0.715–0.870	1.9	0.800	0.712	<0.001 *
Albumin, g/dL	0.736	0.648–0.817	2.5	0.622	0.755	<0.001 *
Lactate/albumin ratio	0.839	0.772–0.898	0.82	0.778	0.799	<0.001 *

Abbreviations: AUC: area under the curve. CI: confidence interval. * Statistically significant (*p* < 0.05). ** Youden index was used to determine the optimal cut-off value.

**Table 4:** A comparison of the baseline clinical parameters of the patients: LAR ≥ 0.82 vs. LAR < 0.82.

Patient Parameters	Overall (*n* = 184) (Mean ± SD)	LAR < 0.82 (*n* = 121) (Mean ± SD)	LAR ≥ 0.82 (*n* = 63) (Mean ± SD)	*p*-Value
APACHE II	22.24 ± 7.05	21.72 ± 7.01	26.29 ± 8.08	0.004 *
SAPS II	42.24 ± 16.56	42.46 ± 15.78	47.78 ± 18.90	0.003 *
Hospital admission day	13.35 ± 14.81	11.74 ± 12.80	16.44 ± 17.76	0.065
ICU admission day	6.49 ± 9.96	5.02 ± 5.42	9.32 ± 14.95	0.030 *
Hemoglobin, g/dL	10.80 ± 2.09	11.00 ± 2.04	10.40 ± 2.19	0.292
Hematocrit, %	33.55 ± 6.10	34.20 ± 5.94	32.20 ± 6.37	0.220
Leukocytes, 10^3^/μL	10.11 ± 7.72	10.70 ± 6.99	9.30 ± 8.97	0.413
Neutrophils, 10^3^/μL	7.94 ± 6.18	8.00 ± 5.34	7.60 ± 7.58	0.988
Platelets, 10^3^/μL	230 ± 108.94	242 ± 105.7	197 ± 104.6	0.002 *
Lymphocytes, 10^3^/μL	1.08 ± 0.89	1.27 ± 0.93	0.84 ± 0.78	0.001 *
Monocytes, 10^3^/μL	0.60 ± 0.60	0.68 ± 0.36	0.41 ± 0.90	0.453
BUN, mg/dL	35 ± 44.73	33 ± 42.2	41 ± 48.1	0.047 *
Creatinine, mg/dL	0.84 ± 1.08	0.80 ± 1.05	0.94 ± 1.14	0.173
CRP, mg/dL	70.9 ± 110.38	58.0 ± 113.7	107.6 ± 103.9	0.419
Albumin, g/dL	2.80 ± 0.67	2.90 ± 0.60	2.40 ± 0.65	<0.001 *
CRP/albumin ratio	29.20 ± 46.11	18.12 ± 42.50	46.80 ± 50.90	0.027 *
Lactate, mmol/L	1.60 ± 1.17	1.30 ± 0.46	2.90 ± 1.16	<0.001 *
Non-survivor at 28 days (*n*/%)	45 (%24.5)	*n*: 10 (%8.3)	*n*: 35 (%55.6)	<0.001 *

Abbreviations: LAR: lactate/albumin ratio. APACHE: acute physiology and chronic health evaluation. SAPS: Simplified Acute Physiology Score. ICU: intensive care unit. BUN: blood ürea nitrogen. CRP: c-reactive protein. * Statistically significant (*p* < 0.05).

**Table 5:** Univariate and multivariate regression analyses of lactate, albumin, and lactate/albumin ratio 28 days following surgery.

	Number of Patients	Univariate OR	95% CI	*p*-Value	Multivariate Adjusted OR	95% CI	*p*-Value
Lactate ≥ 1.9 mmol/L	63 high/121 low	9.90	4.37–22.42	<0.001	2.49	0.56–11.10	0.2310
Albumin ≤ 2.5 g/dL	56 low/128 normal	5.09	2.49–10.41	<0.001	2.93	1.25–6.89	0.0136 *
LAR ≥ 0.82	63 high/121 low	13.88	6.14–31.37	<0.001	4.78	1.09–21.08	0.0386 *

Abbreviations: LAR: lactate/albumin ratio. OR: odds ratio. * Statistically significant (*p* < 0.05).

**Table 6:** Pairwise comparison of ROC curves using DeLong’s test.

Variable Comparison	AUC Difference	*p*-Value
LAR vs. Lactate	0.043	0.006
LAR vs. Albumin	0.103	0.043
Lactate vs. Albumin	0.060	0.348

Abbreviations: LAR: lactate/albumin ratio. AUC: Area Under the Curve.

## Data Availability

The datasets generated and/or analyzed during this study are not publicly available due to privacy but are available from the corresponding author upon reasonable request.

## Abbreviations

The following abbreviations are used in this manuscript:

APACHE	Acute Physiology and Chronic Health Evaluation
AUC	Area under the curve
CI	Confidence interval
CRP	C-reactive protein
TNM	Tumour–node–metastasis
ICU	Intensive care unit
LAR	Lactate/albumin ratio
ROC	Receiver operating characteristic
SAPS	Simplified Acute Physiology Score
SD	Standard deviation
SOFA	Sequential Organ Failure Assessment
CAR	CRP/albumin ratio
BMI	Body Mass Index
BUN	Blood ürea nitrogen
